# Selection of Genetic and Phenotypic Features Associated with Inflammatory Status of Patients on Dialysis Using Relaxed Linear Separability Method

**DOI:** 10.1371/journal.pone.0086630

**Published:** 2014-01-28

**Authors:** Leon Bobrowski, Tomasz Łukaszuk, Bengt Lindholm, Peter Stenvinkel, Olof Heimburger, Jonas Axelsson, Peter Bárány, Juan Jesus Carrero, Abdul Rashid Qureshi, Karin Luttropp, Malgorzata Debowska, Louise Nordfors, Martin Schalling, Jacek Waniewski

**Affiliations:** 1 Institute of Biocybernetics and Biomedical Engineering, Warsaw, Poland; 2 Bialystok University of Technology, Bialystok, Poland; 3 Baxter Novum and Renal Medicine, Karolinska Institutet, Stockholm, Sweden; 4 Center for Molecular Medicine, Karolinska Institutet, Stockholm, Sweden; Harvard Medical School, United States of America

## Abstract

Identification of risk factors in patients with a particular disease can be analyzed in clinical data sets by using feature selection procedures of pattern recognition and data mining methods. The applicability of the relaxed linear separability (*RLS*) method of feature subset selection was checked for high-dimensional and mixed type (genetic and phenotypic) clinical data of patients with end-stage renal disease. The *RLS* method allowed for substantial reduction of the dimensionality through omitting redundant features while maintaining the linear separability of data sets of patients with high and low levels of an inflammatory biomarker. The synergy between genetic and phenotypic features in differentiation between these two subgroups was demonstrated.

## Introduction

Statistical models for analysis of risk factors for a disease or clinical complications, a main focus of medical research, require that the number of patients is larger than the number of variables (factors) to ensure that the statistical significance of the results can be appropriately established. In practice, most studies assess only the influence of each variable separately rather than the combined importance of a set of variables; the former oversimplistic but yet prevailing approach ignores the possibility of interactions between variables or between groups of variables [1]. The obvious need of developing new statistical tools that take into account the extensive interactions between the very large numbers of variables determining biological processes and hence clinical outcomes is increasingly emphasized in modern medical and bioinformatics research.

Medical data sets collected today often have a large number of variables for a relatively low number of patients. This may happen for genetic data sets, where the number of variables (genetic variability, as single nucleotide polymorphism, or gene expression data) can be thousand times greater than the number of patients. Statistical methods are not fully justified in this situation [1]. In such a case, data mining methods can be used instead of, or in addition, to statistical methods [2]. The methods of feature subset selection developed in the scope of data mining play an increasingly important role in the exploratory analysis of multidimensional data sets.

Feature selection methods are used to reduce feature space dimensionality by neglecting features (factors, measurements) that are irrelevant or redundant for the considered problem. Feature selection is a basic step in the complex processes of pattern recognition, data mining and decision making [3], [4]. Interesting examples of applications of feature selection procedures can be found, among others, in bioinformatics [5]. A survey of noteworthy methods of feature selection in the field of pattern recognition is provided in [6].

The feature subset resulting from feature selection procedure should allow building a model on the basis of available learning data sets that can be applied for new problems. In the context of designing such prognostic models, the feature subset selection procedures are expected to produce high prediction accuracy.

We apply here the *relaxed linear separability* (*RLS*) method of feature selection for the analysis of data on clinical and genetic factors related to *inflammation*. These data were obtained from the so called *malnutrition, inflammation and atherosclerosis* (MIA) cohort of incident dialysis patients with end-stage renal disease [7] in whom extensive and detailed phenotyping and genotyping have been performed [8], [9]. The cohort was split into two groups: inflamed patients (as defined by blood levels of C-reactive protein, *CRP*, above median) and non-inflamed patients (as defined by a *CRP* below median). Then, genetic and phenotypic (anthropometric, clinical, biochemical) risk factors that may be associated with the plasma *CRP* levels were identified by exploring the linear separability of the high and low *CRP* patient groups. Particular attention was paid in this work to study the complementary role of genetic and phenotypic feature subsets in differentiation between inflamed and non-inflamed patients.

Four benchmarking feature selection algorithms were selected for the comparisons with *RLS* method on the given clinical data set: 1) *ReliefF*, based on feature ranking procedure proposed by Kononenko [10] as an extension of the *Relief* algorithm [11], 2) *Correlation-based Feature Subset Selection - Sequential Forward* algorithm (*CFS-SF*) [12], 3) *Multiple Support Vector Machine Recursive Feature Elimination* (*mSVM-RFE*) [13] and 4) *Minimum Redundancy Maximum Relevance* (*MRMR*) algorithm [14]. The *CPL* method and four other frequently used classification methods (*RF* (*Random Forests*) [15], *KNN* (*K - Nearest Neighbors*, with K = 5) [3], *SVM* (*Support Vector Machines*) [16], *NBC* (*Naive Bayes Classifier*) [3]) were applied for classification of patients on the basis of the selected features.

## Methods

### Relaxed Linear Separability Method

A detailed description of the *relaxed linear separability* (*RLS*) method as applied in the present study is provided in Appendix S1 together with all the definitions. A brief summary of the method is presented below.

The *RLS* method of feature subset selection is linked to the basic concept of linear separability. The linear separability means possibility of two learning sets separationby a hyperplane [17], [18]. The linear separability notion originated from the perceptron model linked to the beginning of neural networks [19]. Detection and evaluation of linear separability can be carried out efficiently by minimizing the *perceptron criterion function* [3]. This function belongs to the more general class of the *convex and piecewise-linear (CPL)* criterion functions [20].

The *perceptron* criterion function was modified by adding a regularization component for the purpose of the feature subset selection task [20]. The regularization component has similar structure to those used in the *Lasso regression* [21]. The main difference between the *Lasso* and the *RLS* methods is in the types of the basic criterion functions. The basic criterion function used in the *Lasso* method is that of the *least squared* method, whereas the perceptron criterion function and the modified criterion function are used in the *RLS* method. This difference effects the computational techniques used to minimize the criterion functions. The modified criterion function, similarly to the perceptron criterion function, is convex and piecewise-linear (*CPL*). The basis exchange algorithms allow the identification of the minimum of each of these *CPL* criterion functions [22]. The basis exchange algorithms are similar to linear programming and allow to find the optimal solution efficiently even in the case of large, high dimensional learning sets.

The (*RLS*) method of feature subset selection is based on minimization of the modified perceptron criterion function and allows for successive reduction of unnecessary features while preserving the linear separability of the learning sets by increasing the cost parameter in the modified criterion function. The stop criterion for discarding the unnecessary features was based on the cross-validation error (CVE) rate (defined as the average fraction of wrongly classified elements) estimated by the leave-one-out method.

The evaluation of the *RLS* approach was previously carried out with good results both when applied on simulated high dimensional and numerous data sets as well as on benchmarking genetic data sets [18]. For example, the *RLS* method were used for processing the *Breast cancer* data set [23]. The number of features (genes) in this set is equal to 24481. The *RLS* method allowed to select from this set the optimal subset of 12 genes and such linear combination of these genes (*linear key*), which allows to correctly distinguish with 100% accuracy two leaning sets composed of 46 cancer and 51 non-cancer patients.

### Alternative Methods for Feature Selection and Classification

The *RLS* method of feature subset selection involves generation of the sequence of the reduced feature subspaces 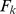 (see Appendix S1, equation 7). The sequence is generated in the deterministic manner through a gradual increase of the cost level 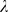 in the minimized criterion function 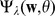 (see Appendix S1, equation 5). In order to determine the best (final) subspace 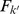 in the sequence an evaluation of the quality of individual subspaces 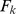 is needed. Traditionally, the quality of the feature subspaces 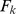 is evaluated through the quality evaluation of the classifiers built in this subspace. Statistical methods for evaluation and comparison of classifiers can be found in [24]. This section presents a few other previous methods of feature selection and classification that were applied for the analysis of the MIA data sets, for comparison of the results, see Results.

Four benchmarking feature selection algorithms were chosen for an experimental comparison with the *RLS* method. One of the selected algorithms, *ReliefF*, is based on feature ranking procedure proposed by Kononenko [10] as an extension of the *Relief* algorithm [11]. The *ReliefF* searches for the nearest objects from different classes and weighs features according to how well they differentiate these objects. The second one is a subset search algorithm denoted as *CFS-SF* (*Correlation-based Feature Subset Selection - Sequential Forward*) [12]. The *CFS-SF* algorithm is based on a correlation measure which evaluates the goodness of a given feature subset by assessing the predictive ability of each feature in the subset and a low degree of correlation between features in the subset. These two feature selection algorithms are considered as “the state of the art” tools for feature selection [4]. The third algorithm, *mSVM-RFE*, is a relatively new idea. It is an extension of the *SVM-RFE* algorithm (*Support Vector Machine Recursive Feature Elimination*). The *SVM-RFE* is an iterative procedure that works backward from an initial set of features. At each round it fits a simple linear *SVM*, ranks the features based on their weights in the *SVM* solution, and eliminates the feature with the lowest weight [25]. Multiple *SVM-RFE* (*mSVM-RFE*) extends this idea by using resampling techniques at each iteration to stabilize the feature rankings [13]. The fourth algorithm *MRMR* (*Minimum Redundancy - Maximum Relevance*) [14] is also a relatively new idea. It bases on feature ranking procedure with special ranking criterion. The position of single feature in the list depends both on its correlation with class and dissimilarity to each feature above it in the ranking.

To compare feature selection algorithms and to evaluate the selected feature subspaces, four frequently used classification methods, beside the *CPL* method, were applied:
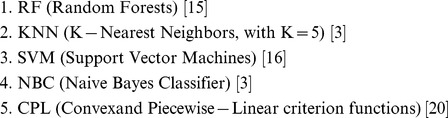
(1)


The four first classifiers (1) were designed by using *Weka*'s implementation [26]. The *Weka*'s implementation of *ReliefF* and *CFS-SF* was used also for the feature selection and cross validation evaluation of designed classifiers. The *R* implementation of *mSVM-RFE* was used (*SVM-RFE* package) [27]. The results of *MRMR* was obtained with the help of the code provided by its author [28]. The *CPL* classifiers based on the search for optimal separating hyperplane 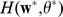 (see Appendix S1, equation 1) through minimization of the *CPL* criterion functions 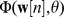 (see Appendix S1, equation 4) was applied using our own implementation. Our own implementation was also used for the *RLS* method of feature selection [18].

### Clinical Data Sets

Two learning sets 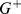 and 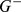 were selected from a cohort of patients with chronic kidney disease, the *MIA* cohort [7]. The set 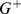 contained 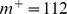 patients 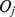 with a high *CRP* levels (above the median value) and the set 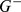 contained 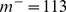 patients 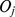 with a low plasma *CRP* levels (below the median value). Each patient 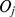 from the learning sets 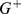 and 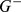 was characterized by numerical results 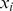 (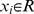) of 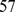 anthropometric or biochemical measurements and by 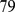 sites of genetic polymorphism (single nucleotide polymorphisms (*SNPs*) or deletions/insertions). The 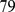 polymorphisms were selected from 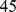 different candidate genes each harboring one to four of these variations. Each site of the genetic polymorphism was characterized by (usually three) binary features 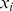 (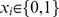), 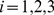, that described three possible genotypes at this site (for example 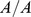, 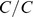, 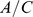). The value one (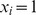) of the binary feature 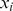 represented the appearance of a particular genotype at the polymorphic site. Thus, each patient 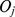 was represented by the 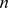-dimensional feature vector 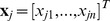, where 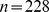 is the total number of features and 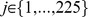 represents the order number (*index*) of a patient 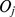 in the cohort of 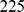 patients. The number of genetic features, 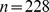, is lower than the expected value of 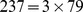 because several genes appeared in the studied population as only one or two genotype forms, i.e., the polymorphism in these genes was not found or was reduced - such cases were coded with less than three binary features. There was also one gene with three alleles and it was coded with five binary features.

These cohort and feature sets were selected from a larger data set and included only those patients for whom at least 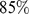 of features were available and those features that were measured for at least 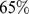 of the patients. In the selected cohort there were still missing data; therefore, for each missing datum, its value for the nearest neighbor in the respective learning set (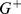 or 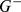) was assigned. The phenotypic and genetic features were considered separately in the procedure of allocating the missing data. In the case of a missing phenotypic feature value, the nearest neighbour was the patient that had the most similar phenotype, whereas for a missing genetic feature value, the nearest neighbour was the patient that had the most similar genotype. The *ce.impute* procedure of *dprep* package of the *R* programming language was used for the substitution of missing values.

During exploration of this database, the computations were performed in feature subspaces 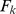 (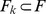) divided in two learning sets 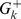 and 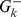. The vectors 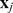 from the set 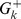 described patients 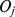 with high plasma *CRP* levels in the feature subspaces 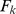. Similarly, the vectors 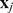 from the set 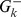 described the patients with low plasma *CRP* levels.

Three basic feature spaces 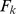 were distinguished as follows:
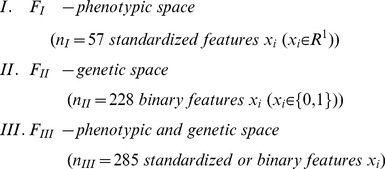
(2)


The *RLS* procedure of feature selection was carried out in each of the basic feature spaces (2) separately.

## Results

**Figure 1:**
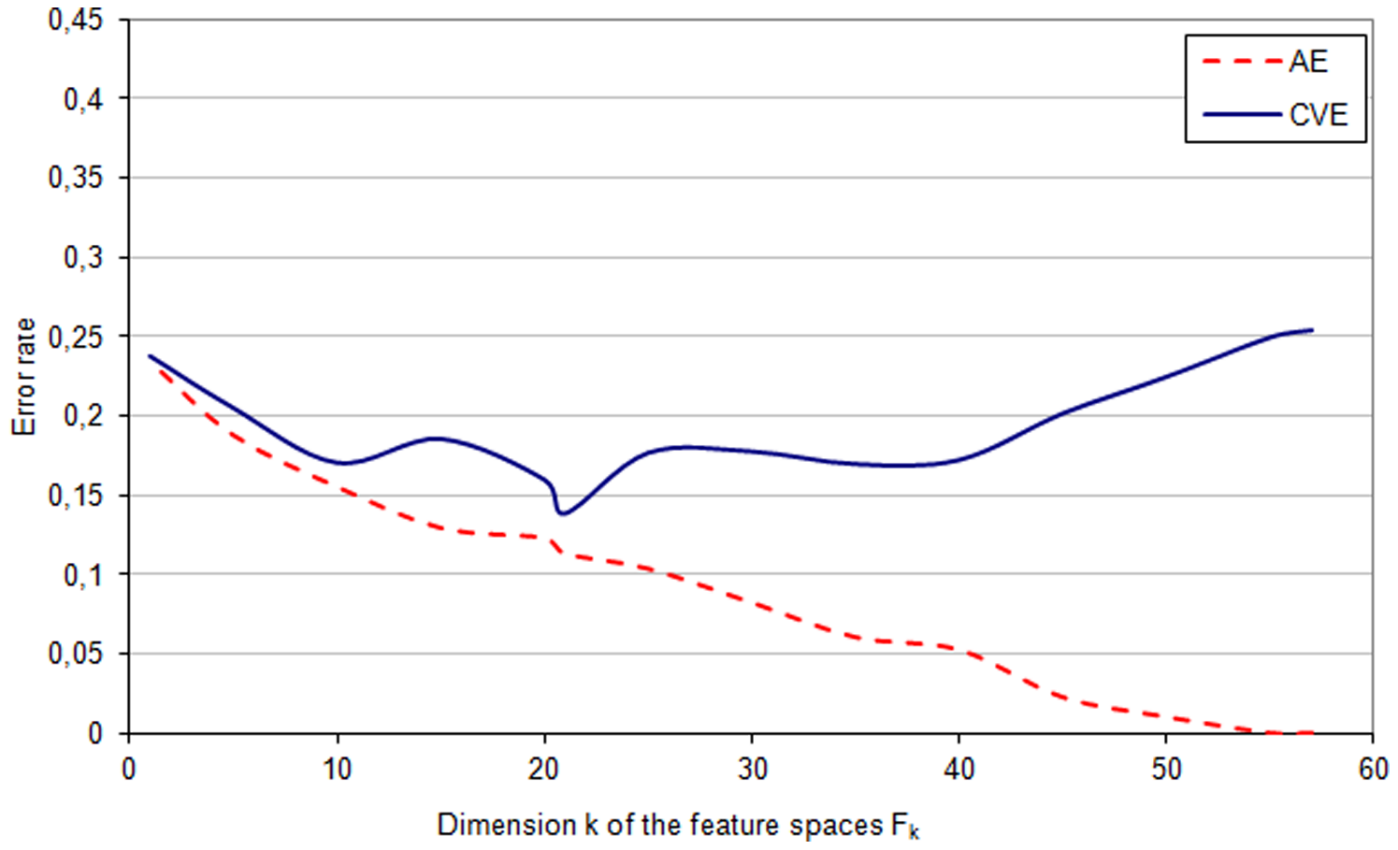
AE and CVE - *phenotypic* space. The apparent error rate (*AE*) and the cross-validation error (*CVE*) in different feature subspaces 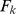 of the *phenotypic* space 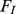.

**Figure 2:**
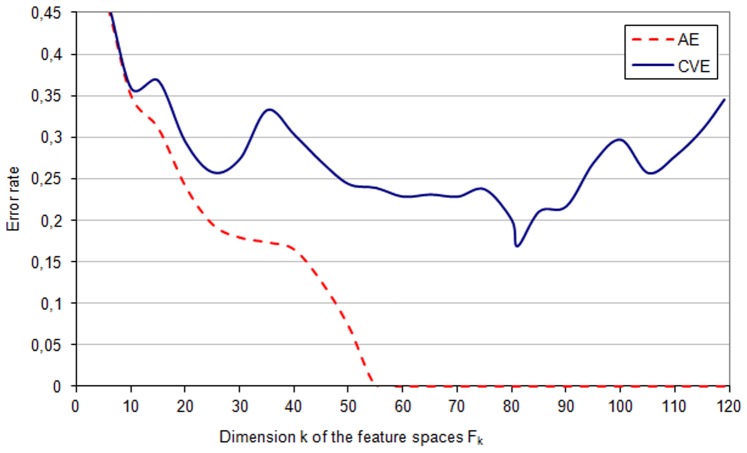
AE and CVE - *genetic* space. The apparent error rate (*AE*) and the cross-validation error (*CVE*) in different feature subspaces 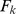 of the *genetic* space 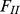.

**Figure 3:**
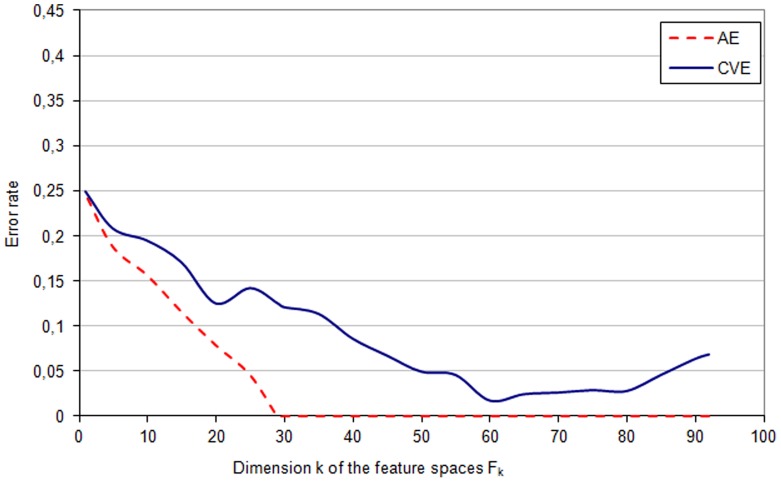
AE and CVE - *phenotypic* and *genetic* space. The apparent error rate (*AE*) and the cross-validation error (*CVE*) in different feature subspaces 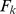 of the *phenotypic* and *genetic* space 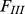.

The apparent error rate 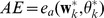 (see Appendix S1, equation 9) and the crossvalidation error rate 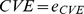 (see Appendix S1, equation 10) of the optimal linear classifier 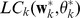 (see Appendix S1, equation 8) as a function of the dimension 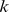 of feature subspaces 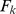 in the sequence (see Appendix S1, equation 7) of the feature spaces 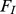, 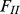 and 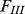, definition (2), are presented in Figures 1–3.

The apparent error rate (*AE*) and the cross-validation error (*CVE*) in feature subspaces 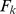 of the *phenotypic* space 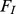 are shown in Figure 1. The lowest value of (*CVE*) equal to 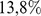 appeared in the feature subspace 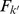 of the dimension 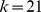. The features that define this subspace 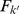 are presented in Table 1. The features listed in Table 1 were ordered according to the absolute values 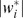 (*factors*) of the components of the optimal weight vector 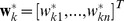.

**Table 1:** Features that define the optimal phenotypic subspace 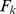 characterized by the lowest cross-validation error (*CVE*), their factor coefficients 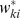 in the minimal value of the criterion function 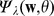 (see Appendix S1, equation 5) and their correlation coefficients with *CRP* plasma concentrations.

Feature	Factor	Pearson’scorrelation	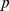 -value
*Serum fibrinogen*	1,478	0,483	0,000
*Plasma iron*	1,066	−0,389	0,000
*Serum ferritin*	1,023	0,238	0,000
*Height*	0,841	0,098	0,141
*Serum interleukin-6*	0,806	0,396	0,000
*Serum creatinine*	−0,778	−0,070	0,298
*White blood cells count*	0,758	0,351	0,000
*Smoking*	0,754	0,106	0,114
*Plasma insulin*	−0,740	0,017	0,796
*Plasma calcium*	−0,657	−0,085	0,201
*Bone mineral density*	−0,493	−0,084	0,212
*Plasma Troponin T*	0,493	0,225	0,001
*Systolic blood pressure*	−0,433	−0,039	0,559
*Handgrip strength*	0,404	−0,064	0,336
*S-triiodothyronine T3*	−0,393	−0,219	0,001
*Plasma uric acid*	0,301	0,093	0,165
*Age*	0,289	0,323	0,000
*Plasma fetuin*	−0,278	−0,120	0,071
*Truncal fat mass*	0,237	0,225	0,001
*Body mass index*	0,237	0,075	0,264
*Glycated hemoglobin*	−0,153	−0,088	0,189

The features listed in Table 1 was identified as the one subset 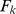 of the feature subspace 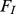. This subset was not composed from the best single features 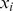. It includes the features that are correlated to *CRP* plasma levels as well as those that are not. Most of the *phenotypic features* listed in Table 1 are in fact expected by medical experts to be related to inflammation but their relative importance is less clear.

Whereas the list of phenotypic features in general appears to be biologically plausible, the ranking of the strength of the association as expressed by the value of the factor coefficient 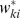 provides novel and potentially important insights into the links between the investigated features and the biomarker selected to represent inflammation, i.e. *CRP*. Thus, some of the identified phenotypic features in Table 1 (i.e., serum fibrinogen, (low) plasma iron, serum ferritin, serum interleukin-6, and white blood cells count) are well established *biomarkers of inflammation*, whereas others are linked to *cardiovascular disease* (plasma troponin T and systolic blood pressure) which is in turn linked to inflammation [29]. However, the negative value for the factor coefficient for systolic blood pressure is an intriguing finding which might reflect that a low blood pressure could be associated with cardiac dysfunction and heart failure, conditions which are known to be associated with inflammation [30]. Other phenotypic features in Table 1 (height, serum creatinine, plasma insulin, plasma calcium, bone mineral density, hand grip strength, S-triiodothyronine T3, plasma uric acid, plasma fetuin, truncal fat mass, body mass index, glycated hemoglobin) are linked to *nutrition* (height, serum creatinine, bone mineral density, hand grip strength, truncal fat mass and body mass index). It is well established that an abnormal nutritional status with protein-energy wasting in this patient population is strongly linked to inflammation [31]. Several features were linked to *hormonal status* or *metabolism* (plasma insulin, plasma calcium, S-triiodothyronine T3, plasma uric acid, plasma fetuin, glycated hemoglobin); in general, relations between these features and inflammation have been described previously, but the relation with plasma calcium is not expected. Finally, high age and smoking are factors which are associated with inflammation.

Feature selection from the *genetic* space 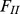 is illustrated in Figure 2. The learning sets 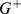 and 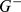 of the space 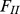 are linearly separable, i.e., the apparent error *AE* is equal to zero. Moreover, the linear separability was preserved during feature reduction from 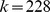 to 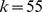. In contrast, the lowest value of the average cross-validation error rate 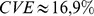 appeared for 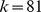. It should be stressed, that the cross-validation procedure does not separate fully those feature subspaces that are linearly separable (Figure 2).

The process of feature selection from the combined *phenotypic* and *genetic* space 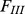 yielded interesting results shown in Figure 3. The linear separability in the combined space 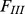 was found in a large range of subspace dimensions from 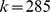 till 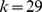. The minimal feature subspace 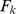 with the linear separability of the learning sets for 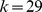 is composed from both *phenotypic* (i.e., clinical, anthropometric and laboratory) features and *genotypes*. The minimal value of the average cross-validation error rate was low: 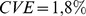. This minimum value appeared at the dimension 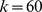 inside the linear separability zone. The optimal feature subspace 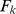 with 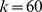 was composed from 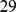 phenotypic features and 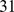 genotypes.

The minimal cross validation error rate in the *phenotypic* space 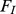 was 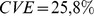 (Figure 1), and the *genetic* space 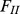 it was 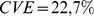 (Figure 2). Combining the *phenotypic* and *genetic* factors (features) resulted in a marked reduction of the CVE error rate to 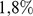. These results indicate that the *phenotypic* and *genetic* factors are not independent and play complementary roles in describing the inflammatory status of the patients in the MIA cohort.

The *confusion matrices* 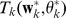 with the mean values obtained by the *leave-one-out* procedure for the *phenotypic* and *genetic* features are presented in Table 2 for a few selected feature subspaces. The lowest error was found for the subspace with dimension 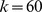 in agreement with the *RLS* method of feature selection.

**Table 2:** The *confusion matrices* 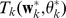 (see Appendix S1, equation 11), for the combined *phenotypic* and *genetic* subspaces 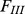 with dimensionalities 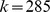, 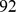, 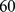, and 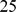.

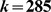	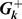	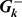
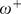	89	23
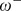	24	89
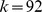	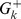	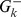
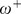	104	8
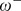	8	105
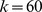	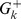	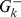
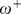	110	2
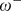	2	111
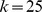	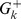	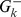
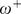	95	17
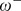	20	93

The optimal parameters 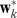 and 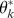 may be used to define the linear (affine) transformation of the feature vectors 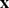 (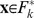) on the one dimensional space 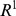:
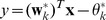
(3)


**Figure 4:**
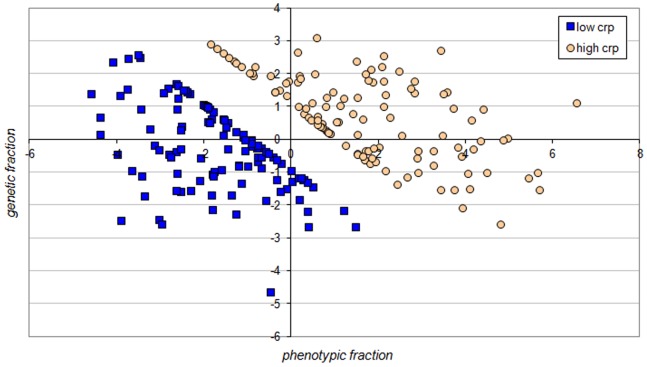
The diagnostic map. Linear separation of the high *CRP* from the low *CRP* patients for the cohort of incident dialysis patients in the optimal feature subspace 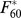 of the phenotypic and genetic space 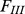.

The above transformation described by equation (3) was applied in designing the *scatter diagram* (*diagnostic map*) showed in Figure 4. The horizontal axis (called *phenotypic fraction*) was obtained by transformation (3) applied for 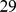 *phenotypic* features that constitute the optimal feature subspaces 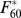 of the *phenotypic* and *genetic* space 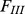. Similarly, the vertical axis (called *genetic fraction*) of the diagram was obtained by transformation (3) applied for 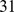 *genetic* features 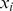 which constitute the optimal feature subspaces 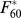.

The *diagnostic map* showed in Figure 4 can be used for diagnosis support. A new patient represented by a feature vector 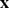 (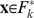) can be situated on the *diagnostic map* as the point 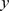 determined by equation (3). If most of the 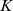 nearest neighbors 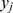 of the point 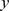 (3) on the map belong to the set 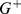 of the high *CRP* patients, then we infer that the new patient is inflamed. If most of the 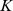 nearest neighbors 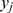 of the point 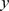 (3) on the map belong to the set 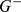 of the low *CRP* patients, then we infer that the new patient is not inflamed. Similar schemes of decision support are called the *K-nearest neighbours* (*KNN*) in the *pattern recognition* or as the *Case Based Reasoning* (*CBR*) scheme [18].

The transformation of the multidimensional feature vectors 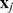 (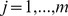) from the learning sets 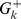 and 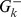 and the feature vector 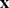 of a currently diagnosed patient on a two-dimensional diagnostic map are aimed at obtaining a *similarity measure* 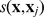 [20]. The measure 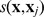 allows for the determination of the similarity between the vector 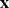, representing a newly diagnosed patient, and the 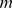 *precedents* (*cases*, *verified examples*) from the learning sets (clinical database). Such scheme of the decision support based on the *diagnostic maps* has been used successfully in the medical diagnosis support system *Hepar* [32].

The performance of *RLS* selection method and *CPL* classifier applied in our study was compared to other selection methods and classifiers (see Section “Alternative methods for feature selection and classification”) using the error rate (fraction of misclassified objects from the test set), *CVE*, evaluated in the *cross-validation* (*leave-one-out*) procedure [3]. The results are presented in Tables 3–5. The methods *CFS-FS* and *mSVM-RFE* alongside with *RLS* select an optimal subset of features and their prediction power can be assessed using different classifiers. In contrast, *ReliefF* and *MRMR* methods are ranking procedures and od not provide any intrinsic criteria for selection of any optimal subset of features. Such criterion need to be chosen separately. For the purpose of comparison of all these methods, the optimal sets of features for *ReliefF* and *MRMR* were determined for each classifier separately as those with minimal *CVE* for the applied classifier. Thus, the optimal set (and number) of features for these two methods can vary with the choice of classifier (see Tables 3–5).

**Table 3:** The cross validation error *CVE* (mean 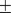 SD) for different classifiers in the *phenotypic* space 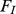 and their subspaces obtained by using five features selection methods (*RLS*, *ReliefF*, *CFS-FS*, *mSVM-RFE*, *MRMR*) and five classifiers (*RF*, *KNN*, *SVM*, *NBC*, *CPL*), see Section “Alternative methods for feature selection and classification”.

Feature selection method	Number offeatures	Classifier				
		RF	KNN	SVM	NBC	CPL
No selection	57	0,231	0,329	0,258	0,302	0,258
		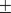 0,422	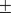 0,470	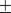 0,437	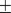 0,459	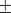 0,437
ReliefF	*	0,173	0,240	0,160	0,240	0,156
		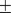 0,379	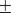 0,428	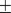 0,367	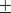 0,428	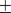 0,362
		(25)	(28)	(26)	(3)	(26)
CFS-FS	15	0,218	0,196	0,178	0,267	0,191
		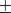 0,413	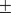 0,397	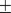 0,382	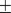 0,442	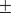 0,393
mSVM-RFE	26	0,200	0,338	0,151	0,231	0,178
		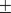 0,400	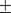 0,473	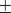 0,358	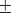 0,422	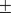 0,382
MRMR	*	0,182	0,182	0,169	0,240	0,173
		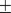 0,387	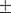 0,387	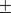 0,375	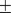 0,427	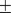 0,379
		(30)	(12)	(11)	(21)	(8)
RLS	21	0,191	0,311	0,156	0,280	0,138
		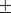 0,393	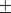 0,463	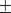 0,362	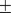 0,449	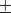 0,345

* ReliefF and MRMR are ranking procedures. The optimal sets of features for these two methods were determined for each classifier separately; the number of features (shown in parentheses) corresponds to the size of the subset of features characterized by the smallest cross validation error for the specific classifier.

**Table 4:** The cross validation error *CVE* (mean 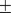 SD) for different classifiers in the *genetic* space 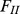 and their subspaces obtained by using five features selection methods (*RLS*, *ReliefF*, *CFS-FS*, *mSVM-RFE*, *MRMR*) and five classifiers (*RF*, *KNN*, *SVM*, *NBC*, *CPL*), see Section “Alternative methods for feature selection and classification”.

Feature selection method	Number offeatures	Classifier				
		RF	KNN	SVM	NBC	CPL
No selection	228	0,502	0,436	0,444	0,493	0,462
		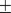 0,500	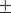 0,496	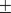 0,497	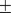 0,500	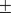 0,499
ReliefF	*	0,338	0,293	0,347	0,369	0,369
		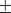 0,473	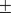 0,455	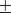 0,476	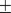 0,483	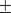 0,483
		(22)	(76)	(82)	(26)	(39)
CFS-FS	3	0,458	0,427	0,427	0,422	0,427
		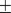 0,498	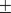 0,495	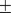 0,495	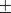 0,494	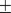 0,495
mSVM-RFE	140	0,48	0,342	0,356	0,458	0,378
		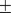 0,500	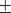 0,474	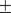 0,478	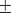 0,498	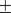 0,485
MRMR	*	0,347	0,333	0,280	0,280	0,276
		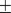 0,476	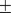 0,471	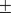 0,449	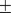 0,449	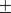 0,447
		(21)	(70)	(38)	(21)	(25)
RLS	81	0,489	0,418	0,338	0,418	0,169
		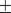 0,500	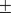 0,483	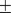 0,473	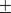 0,493	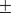 0,375

* ReliefF and MRMR are ranking procedures. The optimal sets of features for these two methods were determined for each classifier separately; the number of features (shown in parentheses) corresponds to the size of the subset of features characterized by the smallest cross validation error for the specific classifier.

**Table 5:** The cross validation error *CVE* (mean 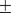 SD) for different classifiers in the *phenotypic* and *genetic*space 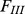 and their subspaces obtained by using five features selection methods (*RLS*, *ReliefF*, *CFS-FS*, *mSVM-RFE*, *MRMR*) and five classifiers (*RF*, *KNN*, *SVM*, *NBC*, *CPL*), see Section “Alternative methods for feature selection and classification”.

Feature selection method	Number offeatures	Classifier				
		RF	KNN	SVM	NBC	CPL
No selection	285	0,293	0,382	0,218	0,293	0,209
		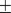 0,455	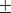 0,486	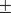 0,413	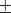 0,455	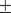 0,407
ReliefF	*	0,191	0,240	0,187	0,200	0,213
		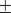 0,393	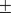 0,427	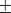 0,390	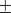 0,400	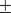 0,410
		(80)	(2)	(54)	(16)	(61)
CFS-FS	15	0,218	0,196	0,178	0,267	0,191
		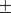 0,413	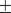 0,397	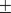 0,382	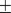 0,442	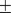 0,393
mSVM-RFE	153	0,262	0,382	0,156	0,302	0,182
		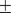 0,440	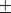 0,486	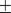 0,362	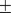 0,459	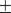 0,386
MRMR	*	0,160	0,267	0,129	0,213	0,156
		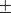 0,367	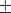 0,442	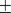 0,335	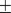 0,410	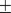 0,362
		(25)	(1)	(44)	(27)	(39)
RLS	60	0,231	0,378	0,018	0,258	0,018
		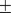 0,422	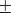 0,485	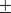 0,132	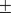 0,437	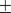 0,132

* ReliefF and MRMR are ranking procedures. The optimal sets of features for these two methods were determined for each classifier separately; the number of features (shown in parentheses) corresponds to the size of the subset of features characterized by the smallest cross validation error for the specific classifier.

All the applied methods of feature selection were able to reduce the initial number of features (Tables 3–5). The highest reduction was obtained by *CFS-FS* method, which substantially outperformed in this respect four other methods. The features selected by *RLS* method provided however the lowest average cross validation error *CVE* for all three feature spaces. Especially low errors of 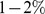 (with standard deviation of 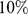) obtained for *RLS* method in the combined phenotypic and genotypic feature space (Table 5) demonstrate its good efficiency. The number of features was reduced in this case five times. For the space of genetic features, only *RLS* selection method combined with *CPL* classifier was able to obtain the low average error around 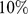, much lower that values of around 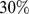 or higher obtained by other selection methods and classifiers (Table 4). In the case of phenotypic features, the five selection methods had a similar performance, but *RLS* method yielded slightly lower errors than the four other methods (Table 3). *MRMR* provided in all three feature spaces lower error values than other methods alternative to *RLS*, especially for *SVM* and *CPL* classifiers; however, the optimal sets of features defined according to the minimal *CVE* value for *MRMR* depended on the selected classifier and this reasult would need further attention and investigation of the scope of these different optimal sets. It is also worth to notice that by allowing for higher errors (similar to those obtained for *CFS-FS* method), one can easily reduce further the number of features selected by *RLS* method as it can be seen in Figures 1–3. Among classifiers, *SVM* and/or *CPL* yielded the lowest errors when combined with *RLS* or *CFS-FS* selection methods. *ReliefF* method worked also well with *RF* and *KNN* classifiers. The errors related to the application of *mSVM-RFE* were similar to those related to *ReliefF* and *CFS-FS* methods (Tables 3 and 4).

The overlap between the features selected by different methods was not high. For example, among the 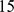 features selected by *CFS-FS* method from the combined phenotypic and genetic features (Table 5), three were shared among all three methods and seven with only one of the two other methods; five features were specific for the *CFS-FS* method. However, the problem of overlapping between features cannot be easily interpreted because many features are more or less correlated and different methods may select different features fromthose that are mutually correlated. Therefore, an additional analysis would be necessary to investigated this problem; however, this is outside the scope of this study.

Among the four applied feature selection methods, *CFS-FS* was the fastest (computation time of the order of 1 sec). *ReliefF* and *MRMR* (together with the selection of optimal set) needed between a few and a few tens of minutes (depending on the applied classifier). The computation time of the *RLS* method was of the order of tens of minutes. The *mSVM-RFE* method had the computation time of about 20 hours. It should be stressed that the relatively long computation time of the *RLS*, *mSVM-RFE*, *ReliefF* and *MRMR* methods was caused mainly by repeated computation in the framework of the cross-validation procedure used by these methods.

## Discussion and Conclusions

Feature selection is an integral - but often implicit - component in statistical analyses. An explicit systematic feature selection process is of value for identifying features that are important for prediction, and for analysis on how these features are related, and furthermore it provides a framework for selecting a subset of relevant features for use in model construction. The most common approach for feature selection in clinical and epidemiological research is based so far on evaluation of the impact of single features [4]. In this approach, the resulting feature subsets are composed of such features (factors) which have the strongest individual influence on the analyzed outcome (in this case inflammation). Such approach is related to the assumption about the independence of the factors. However, in a complex system, such as the living organism, these factors are more often related than not related. The role of particular factors in a living organism depends among others on (time-dependent) environmental factors and internal conditions, and on (permanent) genetic factors. An advantage of the relaxed linear separability (*RLS*) method is that it may identify directly and efficiently a subset of related features that influences the outcome and that it assesses the *combined* effect of these features as prognostic factors. This characteristic of the approach presented here is clearly visible in the dataset of phenotypic features with minimal cross validation error rate, Table 1: this set contains also features that individually do not correlate to the level of *CRP* in plasma, the clinical biomarker used here for discrimination of inflamed and non-inflamed patients.

The *RLS* method of feature selection is based on the minimization of the criterion function 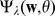 (see Appendix S1, equation 5) for selected values of the cost level 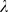 and repeated minimizations of the perceptron criterion function 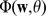 (see Appendix S1, equation 4) in consecutive reduced feature subspaces 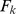 (see Appendix S1, equation 7). The *CPL* criterion function 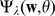 can be defined for different values of the cost level 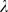 (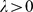) in the same feature space 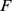. Successive increasing of the parameter 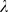 in the function 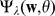 allows to reduce increasing number of features and, as the result, the obtain the descended sequence of feature subspaces 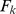. A feasibility of feature subspaces 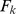 can be evaluated on the basis of the cross validation experiment with the optimal linear classifier 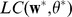 (see Appendix S1, equation 8). The parameters 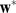 and 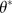 of the optimal classifier are defined on the basis of repeated minimizations of the perceptron criterion function 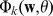 on elements 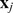 of the learning sets 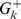 and 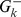 in subspace 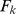.

The application of this method for identifying genetic and phenotypic (anthropometric, clinical and biochemical) risk factors that are associated with inflammation was implemented using a clinical database of patients with chronic kidney disease. A few important properties of the computation results obtained from this cohort can be pointed out. The results show, among others, the scale of the bias of the apparent error (*AE*) estimator (see Appendix S1, equation 9). The bias is illustrated as the difference between the *CVE* curve and the *AE* curve (Figures 1–3). The optimal feature subspace 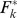 characterized by the lowest *CVE* error rate 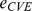 (see Appendix S1, equation 10) cannot be identified on the basis of the apparent error *AE* curve because of this bias. The minimum of the *CVE* rate is clear and narrow for the analysis of genetic data (Figure 2), whereas it is less marked for phenotypic and phenotypic-genetic data sets (Figures 1 and 3) with *CVE* curves fluctuating for a wide range of feature numbers. These two cases may need an analysis of not only the feature space with minimal *CVE* but also the feature spaces with similar, albeit slightly higher *CVE* values. It is also interesting to observe that the lowest values of *CVE* occur for feature subspaces with zero apparent error rate, if genetic and phenotypic-genetic feature spaces are analyzed (Figures 2 and 3), whereas for phenotype feature space the minimum is within the range of subspaces with non-zero apparent error rate (Figure 1).

Working with large medical data bases one meets often the problem of missing data, which was encountered also in our database. The patients with too many features missing and features that are measured for too low number of patients must be excluded. However, with sufficiently many data one can restore missing values by hypothetical values, and in our study this was done by the value of the nearest neighbour, separately for the phenotypic and genotypic features. Another practical problem is the overfitting of the data that happens when many features are studied for a relatively low number of patients, and this problem occurs also in our database: the two sets of patients with different inflammatory status can be linearly separated as indicated by zero apparent error for all features in the case of genetic, phenotypic and combined sets of features (Figures 1–3). Therefore, to provide a more reliable method for identifying the most predictive subset of features, the cross validation error was applied together with the *leave-one-out* procedure. These two problems preclude actually any statistical *proof* of the studied associations between features in our patient populations and the study should be considered rather as an example of *exploratory analysis* for associations that should be further investigated. We hope that our approach can supplement the current methods for analyses of such complex data which are difficult to collect, and, at the same time, represent unique and medically promising sets of data.

An important characteristic of feature selection methods is the predictive power of the selected feature set, as assessed in the present study by cross validation error (Tables 3–5). The *RLS* method combined with *CPL* classifier was of similar effectiveness as some other methods if applied for phenotypic features represented mostly by continuous variables (Table 3), considerably better than all other methods if applied for genetic features represented by discrete (zero - one) variables, Table 4, and much better than all other methods if applied for combined phenotypic and genetic features represented by mixed type mathematical variables (Table 5). Therefore, the *RLS*/*CPL* approach may be considered as a viable and promising tool for analysis of the extent by which the genetic pool, and, especially the combination of genetic variability and phenotypic characteristics of the patient, may associate with selected features in patient populations.

The computational time for our method depends on two factors: 1) the number of cases and features, and 2) the repetition of calculations for the cross-validation method. The actual computing time for personal computer implementations was in the order of tens of minutes, and was longer than for some alternative methods (see Results), but all the computational times were reasonably short for the current research purpose. However, the computation time may be a limitation of the *RLS* method if applied in the future for data bases with huge amount of data and many patients, or both, and the parallelization of the code or the application of main frame computers may be necessary. Our results suggest that the considerably lower prediction errors obtained for our approach compared to those yielded by faster methods, especially for combined genetic and phenotypic data, make such extensions of the code worthwhile.

The comparison between the optimal feature subspaces 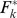 of the three feature spaces (*phenotypic*, *genetic*, *combined*) showed that the combined *phenotypic* and *genetic* subspace can provide a very low *CVE* error rate of 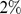 (Figure 3 and Table 5). Such a low error rate opens the possibility for effective computer support of medical diagnosis on the basis of optimal linear combination of selected phenotypic and genetic features. Moreover, an individualization ofdiagnosis and/or therapy can also be considered on the basis of our methods, as, for example, the application of the diagnostic map (Figure 4). Nevertheless, the results of the current study should be considered as hypothesis generating and need to be confirmed in separate evaluations, if possible in another larger group of patients.

## Supporting Information

Appendix S1 **Mathematical foundations of the *RLS* method of feature selection.**
(PDF)
