# Supplementary material for: Selection of Genetic and Phenotypic Features Associated with Inflammatory Status of Patients on Dialysis Using Relaxed Linear Separability Method
Source: PLoS One. 2014 Jan 28;9(1):e86630. doi: 10.1371/journal.pone.0086630 (PMC3904924; doi:10.1371/journal.pone.0086630)
Supplement: Appendix S1 — Mathematical foundations of the RLS method of feature selection. (PDF) [file pone.0086630.s001.pdf]

Appendix S1

Mathematical foundations of the *RLS* method of feature selection

We consider a data set that describes m patients O_j, where j is the order number (index) of the patient O_j in the cohort. Each patient O_j is represented by the n-dimensional feature vector $\mathbf{x}_j = [x_{j1}, ..., x_{jn}]^T$, where n is the total number of features (i.e., clinical characteristics and results of diagnostic tests including genetic polymorphisms). The component x_{ji} of the vector $\mathbf{x}_j$ is the numerical result of the i-th measurement or diagnostic test taken on patient O_j. Both the clinical characteristics and the results of laboratory tests performed in patient O_j can be represented by the components x_{ji} ($x_{ji} \in \{0, 1\}$ *or* $x_{ji} \in R^1$).

Linear separability of two learning sets

The feature vectors $\mathbf{x}_j$ can be considered as points in the *feature space* F. We examine a possibility of linear separation of two groups of patients O_j (for example m^+ healthy patients vs. m^- ill patients) in F and for that purpose two learning sets $G^+ = \{\mathbf{x}_j; j \in J^+\}$ and $G^- = \{\mathbf{x}_j; j \in J^-\}$ where J^+ and J^- are disjoined sets of indices j ($J^+ \cap J^- = \emptyset$), are defined. The set J^+ contains m^+ indices, and the set J^- contains m^- indices.

Definition 1 *The sets G^+ and G^- are linearly separable in the feature space F, if and only if there exist such a weight vector $\mathbf{w} = [w_1, ..., w_n]^T \in R^n$, and threshold $\theta \in R^1$ that $(\forall \mathbf{x}_j \in G^+) : \mathbf{w}^T\mathbf{x}_j > \theta$ and $(\forall \mathbf{x}_j \in G^-) : \mathbf{w}^T\mathbf{x}_j < \theta$.*

The parameters $\mathbf{w}$ and θ define the hyperplane $H(\mathbf{w}, \theta)$ in the feature space F:

$$H(\mathbf{w}, \theta) = \{\mathbf{x} : \mathbf{w}^T\mathbf{x} = \theta\} \tag{1}$$

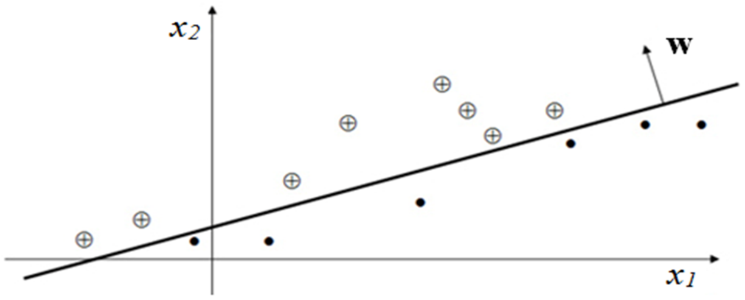

Figure 1. Linearly separable sets. An example of linearly separable sets G^+ (denoted by $\oplus$) and G^- (denoted by $\bullet$) in the two-dimensional feature space F, where $m^+ = 8$ and $m^- = 6$.

If the sets G^+ and G^- are linearly separable then all the elements $\mathbf{x}_j$ of the set G^+ are situated on the *positive side* of the hyperplane $H(\mathbf{w}, \theta)$ ($\mathbf{w}^T\mathbf{x}_j > \theta$) and all the elements of the set G^- are situated on the *negative side* of this hyperplane ($\mathbf{w}^T\mathbf{x}_j < \theta$), see Figure 1.

The concept of *linear separability* is used in the theory of neural networks (*Perceptron* theory) and in pattern recognition methods [1]. Optimal linear classifiers can be designed through exploration of the linear separability of the data subsets [2].

Convex and piecewise-linear criterion functions

The positive penalty function $\varphi^+(\mathbf{w},\theta;\mathbf{x}_j)$ is defined for $\mathbf{x}_j \in G^+$ [1,2]:

$$\begin{array}{l} \varphi^+(\mathbf{w},\theta;\mathbf{x}_j) = 1+\theta-\mathbf{w}^T\mathbf{x}_j \;\; if \;\; \mathbf{w}^T\mathbf{x}_j < \theta+1 \\ \varphi^+(\mathbf{w},\theta;\mathbf{x}_j) = 0 \;\; otherwise \end{array} \tag{2}$$

Similarly, the negative penalty function $\varphi^-(\mathbf{w},\theta;\mathbf{x}_j)$ is defined for $\mathbf{x}_j \in G^-$ [1,2]:

$$\begin{array}{l} \varphi^-(\mathbf{w},\theta;\mathbf{x}_j) = 1-\theta+\mathbf{w}^T\mathbf{x}_j \;\; if \;\; \mathbf{w}^T\mathbf{x}_j > \theta-1 \\ \varphi^-(\mathbf{w},\theta;\mathbf{x}_j) = 0 \;\; otherwise \end{array} \tag{3}$$

The penalty functions $\varphi^+(\mathbf{w},\theta;\mathbf{x}_j)$ and $\varphi^-(\mathbf{w},\theta;\mathbf{x}_j)$ are convex and piecewise-linear (*CPL*) (Figure 2).

The function $\varphi^+(\mathbf{w},\theta;\mathbf{x}_j)$ is equal to zero if the feature vector $x_j \in G^+$ is situated on the positive side of the hyperplane $H(\mathbf{w},\theta)$ and is not too close to it. Similarly, $\varphi^-(\mathbf{w},\theta;\mathbf{x}_j)$ is equal to zero if the vector $\mathbf{x}_j \in G^-$ is situated on the negative side of the hyperplane $H(\mathbf{w},\theta)$ and is not too close to it.

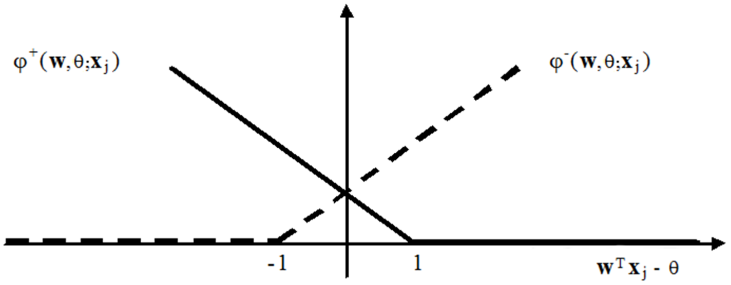

Figure 2. The penalty functions. The penalty functions $\varphi^+(\mathbf{w},\theta;\mathbf{x}_j)$, equation (2), and $\varphi^-(\mathbf{w},\theta;\mathbf{x}_j)$, equation (3).

The *perceptron criterion function* $\Phi(\mathbf{w},\theta)$ is defined on the learning sets G^+ and G^- as the weighted sum of the penalty functions $\varphi^+(\mathbf{w},\theta;\mathbf{x}_j)$ and $\varphi^-(\mathbf{w},\theta;\mathbf{x}_j)$ [1,2]:

$$\Phi(\mathbf{w},\theta) = \sum_{j\in J^+} \alpha_j \varphi^+(\mathbf{w},\theta;\mathbf{x}_j) + \sum_{j\in J^-} \alpha_j \varphi^-(\mathbf{w},\theta;\mathbf{x}_j) \tag{4}$$

where nonnegative parameters α_j can represent *prices* of particular feature vectors $\mathbf{x}_j$. The default values of the parameters α_j are equal to $\alpha_j = 1/(2m^+)$ for $\mathbf{x}_j \in G^+$ and $\alpha_j = 1/(2m^-)$ for $\mathbf{x}_j \in G^-$.

The *optimal parameters* $\mathbf{w}^*$ and θ^* are defined as the values of $\mathbf{w}$ and θ at the minimum $\Phi(\mathbf{w}^*,\theta^*) = \Phi^* \geq 0$ of the criterion function $\Phi(w,\theta)$. It was proved that there is a unique set of optimal parameters $\mathbf{w}^*$ and θ^* and that Φ^* is equal to zero if and only if the sets G^+ and G^- are linearly separable [2].

The perceptron criterion function $\Phi(\mathbf{w},\theta)$ can be defined also on the feature vectors $\mathbf{x}_j$ with reduced dimensionality, i.e. for vectors $\mathbf{x}_j$ that are created with k features instead of the whole set of n features ($\mathbf{x}_j \in F_k,\ k \leq n$). The minimal value Φ^*_k of the function $\Phi_k(\mathbf{w},\theta)$ on F_k can be used as the measures of the degree of the linear separability of the learning sets G^+ and G^- in the reduced feature subspaces F_k. The zero value Φ^*_k indicates that the learning sets G^+ and G^- are linearly separable in the reduced feature subspaces F_k. The reduction of features may increase the value of Φ^*_k [2].

A modified criterion function $\Psi_\lambda(\mathbf{w},\theta)$, which includes additional *CPL* penalty functions based on the absolute values of components $|w_i|$ of vector $\mathbf{w}$ and the *costs* γ_i ($\gamma_i > 0$) of particular features x_i,

was found to be useful for the feature selection [2]:

$$\Psi_\lambda(\mathbf{w},\theta) = \Phi(\mathbf{w},\theta) + \lambda \sum_{i\in\{1,...,n\}} \gamma_i |w_i| \quad (5)$$

where $\lambda \geq 0$ is the *cost level.* The standard values of the parameters γ_i are equal to one.

The modified criterion function $\Psi_\lambda(\mathbf{w},\theta)$ is used in the *relaxed linear separability* (*RLS*) method of feature subset selection [3,4]. The regularization component $\lambda \sum \gamma_i |w_i|$ used in the modified criterion function $\Psi_\lambda(\mathbf{w},\theta)$ is similar to that used in the *Lasso* method developed in the framework of the regression analysis for the model selection [5]. The main difference between the *Lasso* and the *RLS* methods is in the types of the basic criterion functions. The basic criterion function typically used in the *Lasso* method is the *residual sum of squares*, whereas the perceptron criterion function $\Phi(\mathbf{w},\theta)$, equation (4), is used in the *RLS* method. This difference effects the computational techniques applied to minimize of the criterion functions. The criterion function $\Psi_\lambda(\mathbf{w},\theta)$, similarly to the function $\Phi(\mathbf{w},\theta)$ is convex and piecewise-linear (*CPL*). The basis exchange algorithms, which are similar to linear programming, allow the identification of the minimum of the function $\Psi_\lambda(\mathbf{w},\theta)$ or $\Phi(\mathbf{w},\theta)$ even in the case of large, high dimensional learning sets G^+ and G^- [2].

The optimal parameters $\mathbf{w}_\lambda^* = [w_{\lambda 1}^*, ..., w_{\lambda n}^*]^T$ are used in the *feature reduction rule* [3]:

$$(w_{\lambda i}^* = 0) \Rightarrow (the\ feature\ i\ is\ omitted) \quad (6)$$

The reduction of such feature i that is related to the weight $w_{\lambda i}^*$ equal to zero ($w_{\lambda i}^* = 0$) does not change the value of the inner product $(\mathbf{w}_\lambda^*)^T\mathbf{x}_j$. The positive location $(\mathbf{w}_\lambda^*)^T\mathbf{x}_j > \theta_\lambda^*$ or the negative location $(\mathbf{w}_\lambda^*)^T\mathbf{x}_j < \theta_\lambda^*$ of all feature vector $\mathbf{x}_j$ in respect to the optimal separating hyperplane $H(\mathbf{w}_\lambda^*, \theta_\lambda^*)$ remains unchanged.

Relaxed linear separability (*RLS*) method of feature subset selection

The n-dimensional *feature space* F is composed of all the n features x_i from the set $\{x_1, ..., x_n\}$. Feature reduction based on the rule (6) results in appearance of the reduced feature subspaces F_k. The symbol F_k denotes a feature subspace that is composed of k features x_i from the set $\{x_1, ..., x_n\}$. The reduced learning sets G_k^+ and G_k^- are composed from the reduced feature vectors $\mathbf{x}_j$ with the same k features.

Successive increase of the value of the cost level λ in the criterion function $\Psi_\lambda(\mathbf{w},\theta)$ causes reduction of some features x_i and, as the result, allows to generate the descended sequence of feature subspaces F_k with decreased dimensionality ($F_k \supset F_{k-1}$) [2]:

$$F = F_n \rightarrow F_{n-1} \rightarrow ... \rightarrow F_k \rightarrow ... \rightarrow F_1 \quad (7)$$

The reduced feature subspaces F_k are formed from the feature space F as a result of the omission of certain features according to rule (6). The reduced feature vectors $\mathbf{x}_j \in F_k$ are obtained from the feature vectors $\mathbf{x}_j$ in the same manner. The reduction of features may result in the loss of linear separability of the sets G^+ and G^- for some k.

In accordance with the relaxed linear separability (*RLS*) method, the generation of sequence (7) of the feature subspaces F_k is deterministic [3,4]. Each step $F_k \rightarrow F_{k-1}$ is realized by an increase in cost λ values: $\lambda_k \rightarrow \lambda_{k-1} = \lambda_k + \Delta_k$ ($\Delta_k > 0$) in the criterion function $\Psi_\lambda(\mathbf{w},\theta)$. The further increase of the parameter λ allows to reduce the next feature from the reduced vectors in F_k.

The feature reduction process should be stopped by fixing the cost λ at a specific level. The choice of the *stop criterion* of the sequence (7) is a crucial issue in *RLS* method.

RLS stop criterion based on minimal error rate of linear classifier

Two stop criteria for the feature reduction were examined for the *RLS* method. The first of these criteria was based on the error rate of the optimal linear classifiers constructed in the successive feature subspaces F_k [3]. The optimal linear classifier was constructed in each F_k by minimizing the perceptron criterion function $\Phi_k(\mathbf{w}, \theta)$ on the reduced feature vectors $\mathbf{x}_j \in F_k$. The parameters $\mathbf{w}_k^*$ and θ_k^* at the minimum of the criterion function $\Phi_k(\mathbf{w}, \theta)$ determine the following decision rule of the optimal linear classifier $LC_k(\mathbf{w}_k^*, \theta_k^*)$ in the feature subspace F_k:

$$\begin{array}{llll} if & (\mathbf{w}_k^*)^T\mathbf{x} \geq \theta_k^*, & then & \mathbf{x}\ \textit{is allocated to the category}\ \omega^+ \\ if & (\mathbf{w}_k^*)^T\mathbf{x} < \theta_k^*, & then & \mathbf{x}\ \textit{is allocated to the category}\ \omega^- \end{array} \tag{8}$$

where $\mathbf{x} \in F_k$, the category (*class*) ω^+ is represented by m^+ elements of the learning set of reduced vectors G_k^+ and the category ω^- is represented by m^- elements of the learning set of reduced vectors G_k^-.

The quality of the optimal linear classifier $LC_k(\mathbf{w}^*, \theta^*)$, rule (8), can be evaluated using the error estimator (*apparent error rate*) $e_a(\mathbf{w}_k^*, \theta_k^*)$ as the fraction of wrongly classified elements $\mathbf{x}_j$ of the sets G_k^+ and G_k^- in the feature subspace F_k [1]:

$$e_a(\mathbf{w}_k^*, \theta_k^*) = m_a(\mathbf{w}_k^*, \theta_k^*)/m \tag{9}$$

where m is the number of all elements in the sets G_k^+ and G_k^-, and $m_a(\mathbf{w}_k^*, \theta_k^*)$ is the number of elements from these sets wrongly allocated by the rule (8).

If the same data are used for the design of the classifier using rule (8) and for the classifier evaluation, equation (9), the evaluation is biased (too optimistic) [6]. For example, when the learning sets G_k^+ and G_k^- are linearly separable, then all the vectors are correctly classified by the rule (8), and the apparent error (9) is equal to zero. But, as it is often found in practical applications, the error rate evaluated on vectors $\mathbf{x}$ that do not belong to the linearly separable learning sets G_k^+ and G_k^- may be positive.

To reduce the classifier bias, the cross validation procedures can be applied [1,7]. In accordance with the *p-fold cross validation* learning sets G_k^+ and G_k^- are divided into p subsets P_i, where $i = 1, ..., p$. The vectors $\mathbf{x}_j$ contained in $p-1$ subsets P_i are used for the definition of criterion function $\Phi_{k(i)}(\mathbf{w}, \theta)$, equation (4), and for the computation of the optimal parameters $\mathbf{w}_{k(i)}^*$ and $\theta_{k(i)}^*$. The remaining vectors $\mathbf{x}_j$ from the other subset $P_{i'}$ (a test set) are used for the evaluation of error rate $e_i'(\mathbf{w}_{k(i)}^*, \theta_{k(i)}^*)$, equation (9). This evaluation is repeated p times, and during each turn a different p-part $P_{i'}$ is used as the test set. After this, the mean value e_{CVE} of the error rates $e_i'(\mathbf{w}_{k(i)}^*, \theta_{k(i)}^*)$, equation (9), on elements of the test sets $P_{i'}$ is computed.

$$e_{CVE} = \sum_{i=1,\ldots,p} e_i'(\mathbf{w}_{k(i)}^*, \theta_{k(i)}^*)/p \tag{10}$$

The cross validation procedure allows for the use of different vectors for designing the classifier and for its evaluation, and, as the result, to reduce the bias of the error rate estimation. The error rate e_{CVE} estimated during the *cross validation* procedure is called the *cross-validation error* (*CVE*). A special case of the *p-fold cross validation* procedure is called the *leave-one out* procedure, where the number p of the parts P_i is equal to the number m of elements in the sets G_k^+ and G_k^- [8].

Another type of the classifier evaluation is based on the so called *confusion matrix* $T_k(\mathbf{w}_k^*, \theta_k^*)$ [1]:

$$T_k(\mathbf{w}_k^*, \theta_k^*) = \begin{bmatrix} m^{++} & m^{+-} \\ m^{-+} & m^{--} \end{bmatrix} \tag{11}$$

where m^{++} is the number of elements of the set G_k^+ in the feature subspace F_k correctly allocated by the optimal classifier to the category ω^+, and m^{+-} is the number of elements of G_k^+ wrongly allocated to the

category ω^-, m^{--} is the number of elements of the set G_k^- correctly allocated to the category ω^-, and m^{-+} is the number of elements in G_k^- wrongly allocated to the category ω^+.

The confusion matrix $T_k(\mathbf{w}_k^*, \theta_k^*)$ allows to represent different types of errors related to the optimal classifier in the feature subspace F_k. Both the error rate e_{CVE}, equation (10), as well as the confusion matrix $T_k(\mathbf{w}_k^*, \theta_k^*)$, equation (11), can be estimated using the cross validation procedures in different feature subspaces F_k of the sequance (7).

The *cross-validation error rate*, *CVE*, equation (10), of the linear classifier $LC_k(\mathbf{w}^*, \theta^*)$, rule (8), is used in the stop criterion in the present study. The error rate CVE is evaluated for each feature subspaces F_k in the sequence (7). The feature subspace $F_{k'}$ that is linked to the linear classifier $LC_{k'}(\mathbf{w}^*, \theta^*)$ with the lowest error rate e_{CVE} is indicated as the one selected by the classifier. It should be stressed that the estimation of the error rates e_{CVE} is used only for indication of the feature subspace $F_{k'}$, which is considered as having optimal number of features and the features should not be further reduced. The estimation of the error rates $e_{CVE}(\mathbf{w}_k^*, \theta_k^*)$ does not affect in any way the sequence (7) of feature subspaces F_k. In this approach to feature subspace selection, the optimal feature subspace $F_{k'}$ is characterized by the lowest value of e_{CVE}.

RLS stop criterion based on full linear separability

The second type of the stop criterion used in the *RLS* method has been based directly on checking the linear separability of the reduced learning sets G_k^+ and G_k^- [4]. This approach can be useful in the case when the learning sets G^+ and G^- are linearly separable in the full feature spaces F. Let us remark, that the learning sets G^+ and G^- are usually linearly separable in the case when the number of objects, m, is less than the number of the features, n [1]. Such situation appears among others in the case of genetic data sets, where the number n of the features x_i can be thousand times more than the number m of the objects (for example $n = 50000$, $m = 50$). Typically, such learning sets G^+ and G^- are linearly separable.

In accordance with the stop criterion, the feature reduction process is carried out until the linear separability is threatened. The results of the numerical experiments with this stop criterion are described in [4]. The experiments were carried out both with a large synthetic data set as well as with two benchmarking genetic data sets (*Breast cancer* [9] and *Leukemia* [10]).

The synthetic data set contained $m = 1000$ feature vectors with $n = 100$ features. The vectors $\mathbf{x}_j$ were generated randomly in accordance with the multivariate normal distribution $N(0, \Sigma)$ with the covariance matrix Σ equal to the unit matrix I ($\Sigma = I$). The objects $\mathbf{x}_j$ were divided into the learning sets G^+ ($m^+ = 630$ vectors) and G^- ($m^- = 370$ vectors) on the basis of pre-selected linear combination of 10 features x_i (the *linear key*). If the value of this linear key was greater than an assumed threshold, the vector $\mathbf{x}_j$ was attributed to the set G^+. If the value of the linear key was lower than the threshold, the vector $\mathbf{x}_j$ was attributed to the set G^-. Thus, learning sets G^+ and G^- defined in this way were linearly separable. The *RLS* method allowed to skip 90 features x_i while preserving the linear separability of the reduced learning sets G_k^+ and G_k^-, $k \geq 10$, and the linear key of 10 features was correctly rediscovered by the *RLS* method [4].

The *Breast cancer* data set describes 97 patients tested for the presence of the breast cancer [9]. The positive learning set G^+ contains $m^+ = 46$ patients and the negative set G^- contains $m^- = 51$ patients. Each patient is characterized by expression levels of $n = 24481$ genes (features). The *RLS* method allowed to reduce the dimensionality to $n' = 12$ genes while preserving the linear separability of the learning sets G_k^+ and G_k^-. It means that the discovered linear combination of the selected 12 features allowed for full separation of the two subgroups of patients. The computational time on standard personal computer was about 25 min.

The *Leukemia* data set contains expression levels of $n = 7129$ genes in 72 patients [10]. The positive learning set G^+ contains $m^+ = 25$ patients and the negative set G^- contains $m^- = 47$ patients. The *RLS* method allowed to reduce the dimension to three genes while preserving the linear separability of

the learning sets G_k^+ and G_k^-. It means that the discovered linear combination of the three selected genes allowed for 100% correct separation of the two groups of 72 patients.

References

1. Duda OR, Heart PE, Stork DG (2001) Pattern Classification. John Wiley & Sons.

2. Bobrowski L (2005) Data mining based on convex and piecewise linear (CPL) criterion functions (in Polish). Bialystok: Bialystok University of Technology.

3. Bobrowski L, Łukaszuk T (2009) Feature Selection Based on Relaxed Linear Separability. Biocybernetics and Biomedical Engineering 29: 43-59.

4. Bobrowski L, Łukaszuk T (2011) Relaxed Linear Separability (RLS) Approach to Feature (Gene) Subset Selection. In: Xia X, editor, Selected Works in Bioinformatics, InTech. URL `http://www.intechopen.com/articles/show/title/relaxed-linear-separability-rls-approach-to-feature-gene-subset-selection`.

5. Wu TT, Chen YF, Hastie T, Sobel E, Lange K (2009) Genome-wide association analysis by lasso penalized logistic regression. Bioinformatics 25: 714-721.

6. Demšar J (2006) Statistical Comparisons of Classifiers over Multiple Data Sets. Journal of Machine Learning Research 7: 1-30.

7. Kira K, Rendell LA (1992) A practical approach to feature selection. Proceedings of the ninth international workshop on machine learning : 249-256.

8. Lachenbruch PA (1975) Discriminant Analysis. New York: Hafner Press.

9. van 't Veer LJ, Dai H, van de Vijver MJ, He YD, Hart AA, et al. (2002) Gene expression profiling predicts clinical outcome of breast cancer. Nature 415: 530-536.

10. Golub TR, Slonim DK, Tamayo P, Huard C, Gaasenbeek M, et al. (1999) Molecular classification of cancer: class discovery and class prediction by gene expression monitoring. Sciences 286: 531-537.
